# First Demonstration of Clinical *Fusarium* Strains Causing Cross-Kingdom Infections from Humans to Plants

**DOI:** 10.3390/microorganisms8060947

**Published:** 2020-06-23

**Authors:** Thuluz Meza-Menchaca, Rupesh Kumar Singh, Jesús Quiroz-Chávez, Luz María García-Pérez, Norma Rodríguez-Mora, Manuel Soto-Luna, Guadalupe Gastélum-Contreras, Virginia Vanzzini-Zago, Lav Sharma, Francisco Roberto Quiroz-Figueroa

**Affiliations:** 1 Laboratorio de Genómica Humana, Facultad de Medicina, Universidad Veracruzana, Médicos y Odontólogos S/N, Col. Unidad del Bosque, C.P. 91010 Xalapa, Veracruz, Mexico; thuluz@gmail.com; 2 Centro de Química de Vila Real (CQ-VR), Universidade de Trás-os-Montes e Alto Douro, 5000-801 Vila Real, Portugal; rupesh@utad.pt; 3 Instituto Politécnico Nacional, Centro Interdisciplinario de Investigación para el Desarrollo Integral Regional Unidad Sinaloa (CIIDIR-IPN Unidad Sinaloa), Laboratorio de Fitomejoramiento Molecular, Blvd. Juan de Dios Bátiz Paredes no. 250, Col. San Joachín, C.P. 81101 Guasave, Sinaloa, Mexico; yesck_pbs@hotmail.com (J.Q.-C.); luzmgp1@hotmail.com (L.M.G.-P.); rguezmora@hotmail.com (N.R.-M.); che_eucla@hotmail.com (M.S.-L.); gpegastelum21@gmail.com (G.G.-C.); 4 Hospital Para Evitar la Ceguera en México, “Luis Sánchez Bulnes” 11850 Mexico, Mexico; vivanzzini@yahoo.com; 5 Syngenta Ghent Innovation Center, Devgen NV, Technologiepark 30, 9052 Gent-Zwijnaarde, Belgium; lavhere@gmail.com

**Keywords:** keratomycosis, onychomycosis, pathogenicity, horizontal cross-kingdom

## Abstract

Mycotoxins from the *Fusarium* genus are widely known to cause economic losses in crops, as well as high mortalities rates among immunocompromised humans. However, to date, no correlation has been established for the ability of *Fusarium* to cause cross-kingdom infection between plants and humans. The present investigation aims to fill this gap in the literature by examining cross-kingdom infection caused by *Furasium* strains isolated from non-immunocompromised or non-immunosuppressed humans, which were subsequently reinfected in plants and on human tissue. The findings document for the first time cross-kingdom infective events in *Fusarium* species, thus enhancing our existing knowledge of how mycopathogens continue to thrive in different hosts.

## 1. Introduction

The biotic components of any ecosystem are diverse in constitution and dependent on limited and specific resources in order to subsist, adapt, and evolve. Species interact on a broad spectrum, ranging from neutral interaction to lethal parasitism. Elucidating the network of eco-epidemiology is not only vital for understanding disease emergence, but also how it was established and escalated. Other viruses such as bird/swine flu, Ebola, SARS, and recently, nCoV-2019 have been discussed in relation to how cross-species transmission behavior could lead to viral evolution in a new host [1]. Thus, the capacity of a pathogenic organism to be a multi-host infective agent is not restricted to viruses, bacteria, and helminths. Indeed, fungi such as *Aspergillus*, *Penicillium*, and *Fusarium* spp. are able to infect multiple host species [2,3,4,5]. Although infective mechanisms have been observed significantly across species, inter-kingdom infective patterns are very rare. Inter-kingdom pathogens like *Fusarium* as well as others with their cognate hosts are well-known. However, to our knowledge, there is no evidence that the infective pattern is sequentially maintained across the inter-kingdom jump. Interestingly, other diverse parasites in humans such as *Pseudomonas* spp. show the same cross-infecting behavior from plant to animal [6].

The genus *Fusarium* is comprised of diverse and ubiquitous hyaline filamentous fungi that are adaptable to any habitable niche, making them the quintessential opportunistic pathogens [7]. These fungi and their mycotoxins adversely affect approximately 80 economically important crops [8]. However, over the last 30 years, they have emerged as an opportunistic human pathogen, producing lethal systemic infections with a wide range of morbidities in superficial infections [9,10]. This change in epidemiology is likely due to a number of complex factors. Certain strains infect a broad spectrum of host organisms, ranging from plants and insects to humans [3,5,11]. In humans, *Fusarium* can produce fungal keratitis, also known as ocular keratomycosis, which results in severe vision complications. This condition is also a significant cause of surgical intervention in 15%–27% of cases, leading to corneal transplantation, enucleation, removal of eye contents, or even treatment for vision deterioration caused by non-effective drug treatment [12]. Various fungi species can cause this illness, but *Fusarium* is the main causative agent in 37%–50% of fungal keratitis cases [13,14]. Onychomycosis is another human infection caused by *Fusarium* fungi [14]. In plants, *Fusarium* is the most persistent fungi isolated from soil that is associated with vascular invasive mycoses, and its conidia can infect aerial tissue such as corn ears. In maize, *Fusarium* spp. can provoke rots and blight that affect stalk, grain, roots, and seedlings [15,16,17]. Mycotoxins produced by *Fusarium* are a prominent economic issue since they can cause crop loss, in addition to having important animal and human health repercussions [18,19]. *Fusarium* species and their respective strains are rapidly becoming multidrug-resistant [20]. From a health perspective, one of the most harmful *Fusarium*-related diseases is ocular keratomycosis, given the fact that ophthalmic fungal infections represent one of the main etiologic factors of blindness in humans. Although the precise reasons are unknown, the incidence of fungal keratitis has dramatically increased in the last two decades, particularly in countries such as China, India, Brazil, and Mexico [9].

Transmission of pathogens between plants and humans has been hypothesized in the past, but a specific mechanism could not be detected [21]. Fungi are capable of both direct and indirect transmission. Inter-kingdom infective patterns across species is rare. The present study elucidates the ability of clinical samples of *Fusarium* to infect monocotyledonous (corn) or dicotyledonous (*Arabidopsis*) plants as well as human tissue by testing 13 specific fungal keratitis samples from four *Fusarium* species. The findings in the present study are a step forward in clarifying whether there is a cyclical pattern of infection between plants and humans, or whether the infection is only oriented in a plant-to-human direction.

## 2. Materials and Methods

### 2.1. Sample Collection

The human-pathogenic fungal strains initially originated from 13 non-immunocompromised or non-immunosuppressed individuals with keratomycosis, and were acquired by courtesy of the hospital staff at “El Hospital para Prevenir la Ceguera en México, Luis Sánchez Bulnes” between January 2013 and August 2016. Patients were registered from nine states of Mexico with variable age and economic activity (Table 1). In addition, to isolate the potential causative agent of keratitis, eye rub from infected eyes were taken with sterile hyssop and cultured on Sabouraud Dextrose Emmons agar medium agar (SGA; Difco, Detroit, MI, USA). Monoconidia cultures were obtained by serial dilution in Spezieller Nährstoffarmer agar medium (SNA) with a 1-cm^2^ filter paper at 37°C, as described earlier [8]. Colonies were observed growing during the first 48–72 h. The species were identified morphologically on microculture, as in a previous study [22].

### 2.2. Detached in-vitro Tissue Assay

#### 2.2.1. Surface Disinfection of Seeds

Maize seeds were surface disinfected by sonication (ultrasonic bath 2.8L, Fisher Scientific) in sterile distilled water with Tween 20 (0.1% *v/v*) for 5 min. Subsequently, seeds were immersed in 1.5% (*v/v*) sodium hypochlorite (NaOCl) at 52°C for 20 min (Thermobath FE-377, Felisa), followed by rinsing three times in sterile distilled water, and air dried in a Class II Type A2 Biological Safety Cabinet (Herasafe KS, Thermo Scientific, Langenselbold, Germany) and grown in culture room in Magenta™ vessel (no. cat V8505, Sigma, MO, USA) with sterile sand.

#### 2.2.2. Conidia Suspensions and Inoculation of Tissue by *Fusarium*

**Figure 1:**
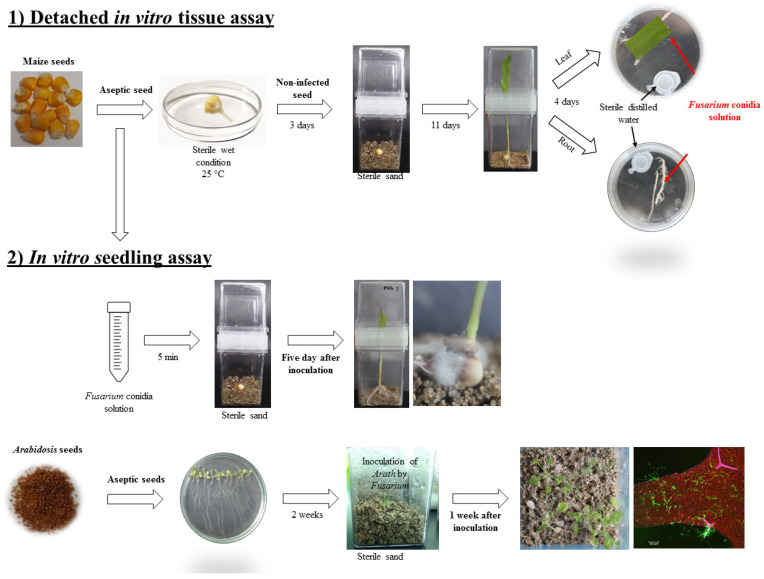
Diagrammatic representation of strategy to evaluate the infective capacity of fungi on plants.

The *Fusarium* strains (Table 1) were cultivated in the SNA medium with a 1-cm^2^ filter paper [8] supplemented with neomycin (0.12 mg/mL) and streptomycin (1 µg/mL), and cultured at 25 ± 2°C for 7 days [23]. Conidia were harvested by adding 5 mL of sterile saline solution (0.8% (*w/v*) sodium chloride) to the culture medium with gentle shaking. The conidia quantification was performed using a Neubauer chamber (Hausser Scientific, Horsham, PA, USA) and a light microscope (B-383-M11, Optika, Ponteranica, Italy) and by CFU on PDA plates. The conidia suspension was prepared with the final concentration of inoculation of 1 × 10^6^ CFU/mL. Leaves and roots were collected from 2 weeks old in-vitro grown maize plants with no visible fungi contamination [24] for detached tissue assays. The tissues were inoculated with 200 µL of conidia solution and incubated at 25°C in a wet chamber and photographed after 5 days post-inoculation (dpi) (Figure 1).

### 2.3. In vitro Seedling Assay

#### 2.3.1. Surface Disinfection of Seeds

The procedure for maize seed surface disinfection was similar to the detached tissue assay. Columbia-0 *Arabidopsis* seeds were surface sterilized for 5 min with 1.25% (*v/v*) NaOCl containing 0.1% (*v/v*) Triton X-100 for four min then rinsed four times with sterile water and sown in 0.1% (*w/v*) sterile agar. All seeds were stratified at 4°C for 3 days and then planted on Petri dishes containing 0.5× Murashigue and Skoog medium [25] supplemented with 0.5% (*w/v*) sucrose and 1% (*w/v*) agar (pH 5.8).

#### 2.3.2. Inoculation of Maize and *Arabidopsis* Seeds by *Fusarium*

Surface-disinfected maize seeds were immersed in the conidia suspension for five minutes, and planted in in vitro culture container with sand, and cultivated at 25 ± 2°C for 5 days. In the case of *Arabidopsis*, in vitro plants 2 weeks old were transferred into in vitro culture container with fertilized sand and inoculated with 200 uL of work suspensions and incubated at 25 ± 2°C for 7 days. Before every assay, both sand and fertilizer were autoclaved at 120°C for 60 min. Photographs were recorded at 5 and 7 dpi for maize and *Arabidopsis*, respectively (Figure 1).

### 2.4. Human Onychomycosis Assay

**Figure 2:**
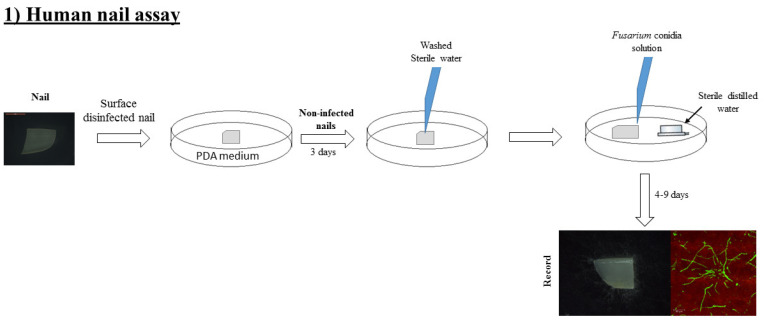
Diagrammatic presentation of strategy to evaluate the infective capacity of fungi to cause onychomycosis.

Small pieces of nails (ca. 5 mm) without polish were washed with 1.25% (*v/v*) NaOCl containing 0.1% (*v/v*) Triton X-100 for 5 min. Nails were rinsed three times with sterile water and incubated for 3 days on PDA at 25 ± 2°C. The non-infected nails were washed three times with sterile water to eliminate any adhered PDA fragments. Nails were placed in a wet chamber (Petri dishes) and the nail edge was infected with 100 µL of work conidia solution. Nails were observed for visible fungal growth from the fourth day onwards until the ninth day (Figure 2).

### 2.5. Fusarium Detection by Confocal Microscopy

In order to visualize *Fusarium* spp. on the nails, samples were stained with WGA-Alexa Fluor^®^ 488 conjugate (W11261, Life Technologies; CA USA), which binds to the chitin molecules on the fungal cell wall [26]. Samples were incubated for 30 min at room temperature in 1× PBS buffer (137 mM NaCl, 10 mM phosphate, 2.7 mM KCl, pH 7.4) supplemented with 1 ng/μL WGA. For visualization, stained nails were placed on a microscope slide and covered with a glass coverslip. Confocal microscopy (Leica TCS SP5 X) was used with the white laser for 499 nm excitation wavelength and emission ranges of 512–526 nm for WGA (green fluorescence) and 632–739 nm to get the autofluorescence signal (red fluorescence).

## 3. Results

Although the cross-kingdom infective capacity of *Fusarium* has been hypothesized, there is no evidence of any sequential human–plant–human tissue reinfection. In order to better understand the broad infectious properties of *Fusarium* spp., the present study assessed the ability of *Fusarium* isolates from human keratomycosis to infect monocot (maize) and dicot (*Arabidopsis*) plants, as well as human tissue in the form of nails. First, the *Fusarium* genotypes were isolated from 15 fungal keratitis samples, initially taken from patients at the “El Hospital para Prevenir la Ceguera en México, Luis Sánchez Bulnes” between January 2013 and August 2016. Each sample was taken from the eye of a non-immunologically compromised patient. Patients came from nine Mexican states and had diverse occupations. Patient age was not restricted to any specific range, but 87.5% were middle-aged adults or older (Figure S1).

**Figure 3:**
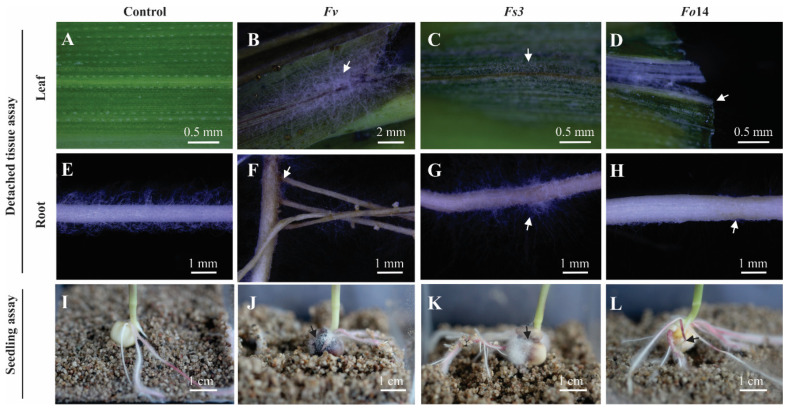
*Fusarium* spp. isolated from human keratitis conserve their plant infective capacity. Detached leaf assay: (**A**) control non-infected leaf; (**B**–**D**) leaves infected by isolated *Fusarium* strains *Fv, Fs*3, and *Fo*14, respectively. Detached root assay: (**E**) control non-infected root; (**F**–**H**) roots infected by isolated *Fusarium* strains *Fv*, *Fs*3, and *Fo*14, respectively. In both detached tissues, leaves and roots were imaged 12 days after infection. Seedling assay: (**I**) control non-infected seedling; (**J**–**L**) seedlings from free-infected seeds inoculated with isolated *Fusarium* strains *Fv*, *Fs*3, and *Fo*14, respectively. Seedlings were imaged 5 days after infection. Samples in the detached tissue and seedling growth assays were under sterile in vitro conditions. Arrows show the sites with abundance fungi growth or damaged tissue. *Fv*: *Fusarium verticillioides*; *Fs*3: *Fusarium solani* isolate no. 3; *Fo*14: *Fusarium oxysporum* isolate no. 14.

In the first stage, we evaluated the infective capacity of *Fusarium* isolates from patients with keratitis to infect plants using two assays. (1) The first assay consisted of the inoculated tissue of detached leaves and root maize seedlings, placed in wet chamber Petri dishes. (2) In the second assay, whole maize and *Arabidopsis* seedlings were inoculated in vitro while still alive. *Fusarium verticillioides*, which infects corn plants was used as a positive control [23,24]. In both experiments, *Fusarium* conidia germinated, colonized the detached tissues, and deterred the growth of in-vivo seedlings (Figure 3). These results demonstrate that *Fusarium* spp. from keratitis patients conserve their infective capacity during the cross-kingdom reinfection jump from humans to plants.

**Figure 4:**
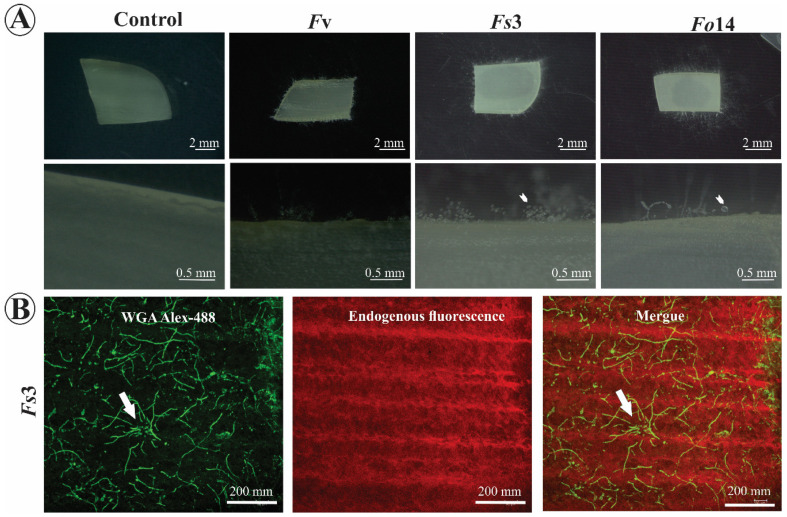
*Fusarium* isolates are a potential causative agent of onychomycosis in human tissue. (**A**) Macroscopic (upper panels) and microscopic (lower panels) imaging of human nails inoculated with *Fv*, *Fs*3, or *Fo*14. (**B**) Colonization of a human nail (which emits endogenous red fluorescence) by *Fusarium Fs*3 isolate (green fluorescence). WGA Alexa-488 was used to detect fungal hyphae in the colonized nail (see ‘Merge’ image). All photographs (**A**) and scans (**B**) were taken 4 days after inoculation in a wet chamber. *Fv*: *Fusarium verticillioides*; *Fs*3: *Fusarium solani* isolate no. 3; *Fo*14: *Fusarium oxysporum* isolate no. 14.

In the second stage, we evaluated the capacity of *Fusarium* strains to back-infect human tissue. In this experiment, the capacity of the isolates to cause onychomycosis was assessed, using human nail samples (Figure 4). Observations suggested that every strain that infected maize and *Arabidopsis* was also able to back-infect human nails. After 4 days of inoculation, *Fusarium* was well established (Figure 4A) and exudates were observed (arrowheads, Figure 4A). Due to the rough surface of the nails, *Fusarium* was able to colonize and use the keratin in them as a growth substrate (arrow, Figure 4B). These results, together with the literature [14], indicate that *Fusarium* might cause onychomycosis in humans. Even though *Fusarium* has been sampled and well-studied in immune-compromised patients [12], the species found in this study were obtained from non-immune-compromised patients, suggesting that the pathogen reached the patients’ eyes by ocular injury.

This study’s findings show that *Fusarium*-infective agents may alter human health, and that their infective capacity to colonize human tissue and plants and to back-infect human tissue (nails) is intact.

## 4. Discussion

There have been several species identified to date that infect both plants and animals. These species range from the extreme, such as *Agrobacterium tumefaciens*, which infects fungi, plants, and animals [27], to the more common species that co-infect plants and animals, including *Aspergillus fumigatus*, *Pleurostomophora richardsiae*, *Pseudomonas aeruginosa*, *Pythium insidiosum*, *Rhizopus oryzae*, *Sporothrix schenckii*, *Staphylococcus aureus*, and *Trichoderma longibrachiatum* [28]. Studies on the infection mechanism in plant hosts, particularly at the immune system level, have revealed that certain features are shared within animal hosts [29]. It is commonly accepted that in plant–animal co-infections, the pathogen is dependent on its ability to recruit iron from the host or environment [30]. Previous studies on *Fusarium* have tried to establish the cross-kingdom pathogenicity between plants, mammals, and insects [31,32,33]; however, to our knowledge, the present study is the first report to demonstrate this infective property between plants and humans.

*Fusarium* is one cause of disease in a wide variety of crops as maize, although it does not produce the same lethality in animals as it does in plants [34]. This might be due to differences between the two kingdoms during pattern recognition by the receptors of innate immune cells [35], which could allow *Fusarium* to evade the host’s immune defenses. One important difference is that the main components of a blood circulatory system, i.e., macrophages, neutrophils, and dendritic cells are not found in plants. In this sense, the pathogenic outcome in both kingdoms can range from lethal to non-lethal outcomes. This may also cause a different clinical result, resulting in a distinct disease with completely different consequences. Taking this into account, there may be two different diseases produced by the same microbial pathogen, which intriguingly appears to contradict to Koch’s third postulate [36]. Just as the well-known evidence of exemptions to Koch’s postulates, it shows that not all parasites can be isolated in artificial media, single isolated pathogens could also produce different symptoms depending on the nature of the host and tissue. A very clear example of this is the fact that *Salmonella* spp. can live within plants without producing any lethal effects, while in animals it is fatal. Although *Fusarium* rarely causes a disease harmful enough to lead to mortality in healthy humans, it can be lethal for plant species. It will be interesting in the future to investigate the similarities between how pathogens escape in order to establish disease. The present research may also facilitate our understanding of how some host species survive an infection while others perish from the same pathogen.

In summary, we investigated whether *Fusarium* species isolated from keratomycosis human patients, which are normally pathogens of plants, conserve their infective capacity to re-infect plants and other human tissue. We demonstrated that *Fusarium* spp. conserves their infective mechanism for colonizing human tissue and plants and for back-infecting other human tissue, such as nails. Our results also found a new exemption to Koch’s third postulate, as the same fungal pathogen was seen to produce two different diseases. This work could serve as a reference for demonstrating cyclic *Fusarium* reinfections between plants and humans. It thus suggests that the infective mechanism of *Fusarium* could be conserved. Further, Omics studies will help to elucidate how *Fusarium* changes its infective mechanism (gene expression and physiology) to adapt to hosts from a different kingdom.

## Figures and Tables

**Table 1:** Profiles of keratitis patients.

Date	Lab ID	Symptomatology	Gender/Age	Patient Location	Occupation	Species
10/01/2013	21564	Ocular trauma	F/75	Tuzamapan, Puebla	Housewife	*Fusarium* *solani*
19/02/2013	21791	DM 10 years, cornea trauma	M/73	Zacatecas	Farmer	*Fusarium* *dimerum*
20/02/2013	21797	Ocular trauma	M/28	Puebla	Farmer	*Fusarium* *solani*
06/03/2013	21890	Ocular trauma	F/32	Quintana Roo	Housewife	*Fusarium* *solani*
08/04/2013	22083	Ocular trauma	M/76	Mexico city	Worker	*Fusarium* *solani*
18/06/2013	22503	Ocular trauma	M/7	Veracruz, Ver	Student	*Fusarium* *solani*
19/08/2013	22869	Insidious, pain+++, immune ring	F/41	La trinitaria Chiapas	Housewife	*Fusarium* *dimerum*
23/12/2013	23544	Insidious, pain++	M/47	AltamiraTamaulipas	Worker	*Fusarium* *solani*
17/02/2014	23813	Ocular trauma	M/41	Mexico city	Builder	*Fusarium* *solani*
20/08/2014	24810	Ocular trauma	M/49	Durango Durango	Farmer	*Fusarium* *solani*
30/01/2015	25704	Insidious, pain	F/34	Acapulco Guerrero	Housewife	*Fusarium* *solani*
27/04/2015	26256	Ocular trauma	F/30	Mexico City	Housewife	*Fusarium* *dimerum*
18/08/2016	28615	Ocular trauma	M/62	Puebla	Farmer	*Fusarium* *oxysporum*

Abbreviations used: DM: diabetes mellitus; pain+++: intense pain; Insidious: patient did not feel any trauma or damage. Patients did not have any immunodeficiencies. F: female; M: male.

## Supplementary Materials

The following are available online at https://www.mdpi.com/2076-2607/8/6/947/s1, Figure S1. Summary of patient characteristics infected by *Fusarium keratitis*. (A) The Mexican states involved in this study, with percentages of keratitis for each. (B) Distribution of *Fusarium* keratitis by gender. (C) Distribution of patient occupations. (D) Distribution of patient age groups.
