# Supplementary figures and images for: First Demonstration of Clinical Fusarium Strains Causing Cross-Kingdom Infections from Humans to Plants
Source: Microorganisms. 2020 Jun 23;8(6):947. doi: 10.3390/microorganisms8060947 (PMC7356758; doi:10.3390/microorganisms8060947)

**A**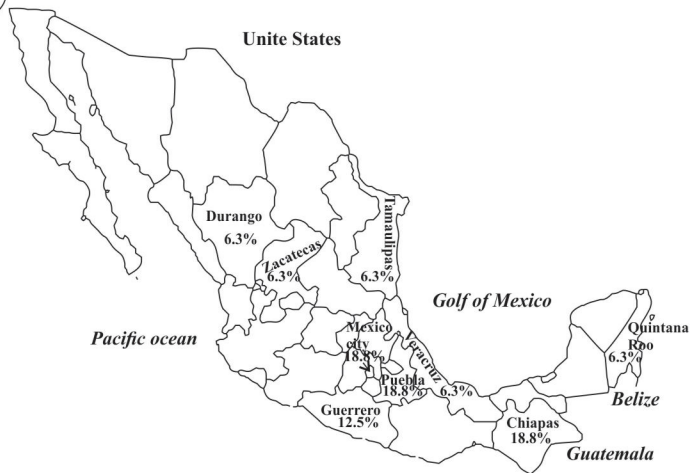**B**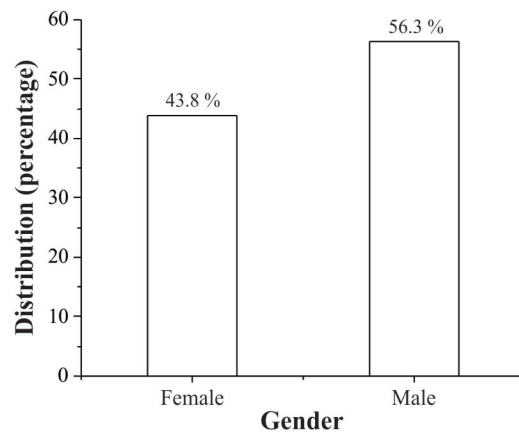**C**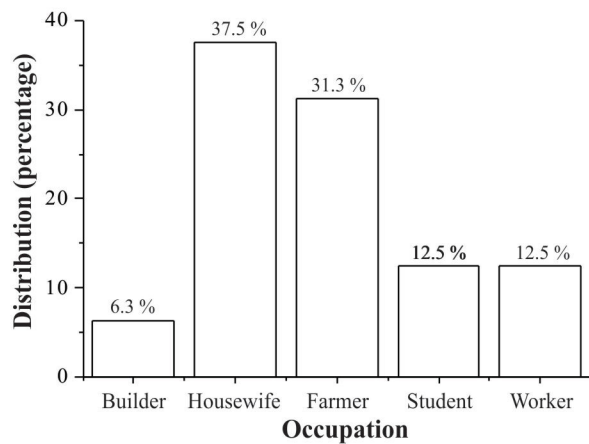**D**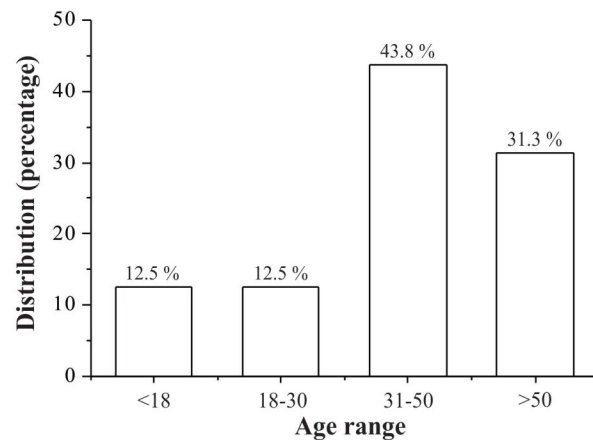

Supplement: Supplementary file 1 [file microorganisms-08-00947-s001.pdf]
